# A Medical Paradigm Shift in Society 5.0: Implementation of a Smartphone App-based Dry Eye Diagnosis Assistance Software as a Medical Device

**DOI:** 10.14789/jmj.JMJ24-0018-P

**Published:** 2024-10-15

**Authors:** TAKENORI INOMATA, JAEMYOUNG SUNG, YUICHI OKUMURA, KEN NAGINO, AKIE MIDORIKAWA-INOMATA, ATSUKO EGUCHI, KUNIHIKO HIROSAWA, YASUTSUGU AKASAKI, TIANXIANG HUANG, YUKI MOROOKA, HIROYUKI KOBAYASHI, SHINTARO NAKAO

**Affiliations:** 1 Juntendo University Graduate School of Medicine, Department of Ophthalmology, Tokyo, Japan; 1 Juntendo University Graduate School of Medicine, Department of Ophthalmology, Tokyo, Japan; 2 Juntendo University Graduate School of Medicine, Department of Hospital Administration, Tokyo, Japan; 2 Juntendo University Graduate School of Medicine, Department of Hospital Administration, Tokyo, Japan; 3 Juntendo University Graduate School of Medicine, AI Incubation Farm, Tokyo, Japan; 3 Juntendo University Graduate School of Medicine, AI Incubation Farm, Tokyo, Japan; 4 Juntendo University Graduate School of Medicine, Department of Telemedicine and Mobile Health, Tokyo, Japan; 4 Juntendo University Graduate School of Medicine, Department of Telemedicine and Mobile Health, Tokyo, Japan

**Keywords:** dry eye, software as medical device, digital therapeutics, mobile health, smartphone application

## Abstract

Society 5.0, proposed as part of the 5th Science and Technology Basic Plan by Japan's National Institute of Advanced Industrial Science and Technology, is a human-centered society where cyberspace and physical space are integrated, to resolve social challenges and promote economic growth. In Society 5.0, medicine will undergo extensive digital transformation (DX), and digital health technology is expected to expand markedly, becoming part of routine clinical practice. Prompt diagnosis of dry eye disease (DED) and uninterrupted monitoring of such patients with healthcare barriers is currently an unmet need. DX of DED evaluation and management can boost the current quality of DED care. Software as Medical Devices (SaMDs), i.e., software programs developed through evidence-based research to provide diagnostic, therapeutic, and preventive services, and particularly medical devices based on smartphone applications (apps), have attracted attention. We have striven to actualize the DX of ophthalmic care and evaluation, denoted by our ongoing development of SaMDs to assist DED diagnosis. To illustrate healthcare using the Internet of Medical Things, we here present the research and development process of our smartphone app-based SaMD for DED diagnosis assistance.

## A medical paradigm shift in Society 5.0

Society 5.0, proposed by Japan's National Institute of Advanced Industrial Science and Technology, is envisioned as a human-centered society with integration of cyberspace and physical space, aimed at resolving social challenges and promoting economic growth^1, 2)^. This new societal structure follows the preceding societal structures, characterized in order of Society 1.0 through 4.0, as hunting, agriculture, industry, and information- oriented societies.

The most notable characteristic of medicine in the era of Society 5.0 is the potential to implement the P4 principles (predictive, preventive, personalized, and participatory) of medicine through highly data-driven biomedical sciences, which was previously impossible^2, 3)^. These data-driven approaches are enabled by the rapid expansion of personalized, real-time medical big data─such as symptomology, digital biomarkers, and lifestyle information, which are accessed through commonplace sensors on smart devices and the Internet of Medical Things (IoMT)─as well as the advances in the artificial intelligence (AI) field, which can effectively analyze the ever-growing medical big data^2, 3)^.

In the current IoMT, smart devices are utilized as a telemedicine platform. Smart wearable devices provide real-time monitoring of digital biomarkers, and virtual reality (VR) devices assist in patient education and treatment, with satisfactory results in clinical practice. Additionally, rapid advances in AI have expanded the use of IoMT devices and medical big data, including comprehensive, personalized digital biodata, such as multi-omics data, to provide individualized regimens^3-6)^. Although the concept of P4 medicine was proposed over two decades ago^7)^, we have now entered a phase where technology can support practical implementation of P4 medicine.

In Japan alone, over 20 AI-based software as medical devices (SaMDs) were approved by the end of 2023, and IoMT utilization has expanded. In other nations, robotic arms, remote surgical systems using VR headsets, and surgical assistive devices based on augmented reality lenses are being developed and submitted for FDA approval^8, 9)^. Interestingly, the healthcare efficacy brought on by the IoMT through better resource allocation, reduced spending, improved profitability in hospital administration, and adjustments in long physician working hours may help to resolve issues related to the aging society, rising medical expenditures, and workforce needs, all of which are being addressed as part of the “Physician Work System Reform,” which was initiated in Japan in April 2024.

Our group has been making efforts to actualize the digital transformation (DX) of ophthalmic evaluation and care, as exemplified by our ongoing development of SaMDs to assist in diagnosis of dry eye disease (DED)^10)^. Herein, as an illustration of healthcare using the IoMT, we discuss the research and development process of our smartphone application (app)-based SaMD for DED diagnosis.

## Smartphone apps in ophthalmology

Global healthcare is undergoing increasingly rapid DX, partially due to the growth in information and communication technology (ICT) and the IoMT sector^11)^, with numerous medical facilities providing digital services through smartphone apps, email communications, chatbots, and voice assistants^12-15)^. With the rapid increase in the prevalence and penetration of smartphones since the first iPhone release in 2008, healthcare providers have attempted to use smartphones as a platform for providing medical services^16)^. As new sensors and functions are increasingly commonly being built into smartphones as a default, access to medically relevant data obtained without requiring conscious input (i.e., physical status, lifestyle patterns, and sleep cycles) has increased dramatically^16-18)^. By incorporating passively collected data into reports that are actively provided by users through direct input (i.e., current symptoms, demographics, and medical history), early disease detection (based on individualized pre-test probability), improved monitoring of recurrence or exacerbations, promotion of positive behavioral changes and adherence to prescribed regimens may become possible^12, 16, 19)^.

Ophthalmology is enjoying a fast-paced implementation of remote care, and we expect that as new research in mobile health (mHealth) continues, ophthalmic care practices will change significantly^20)^. Several smartphone apps related to ophthalmic evaluation have been released, including those for visual acuity evaluation, diabetic retinopathy screening, and assistance in ocular disease diagnosis^21, 22)^. mHealth apps show promise for the early detection and treatment of ocular pathologies, particularly for patients who may remain undiagnosed because of healthcare barriers^11)^. We reported on 48 ophthalmic smartphone apps in a systematic review published in May 2023^23)^. Seventeen of these apps were for ophthalmic evaluation; 13 for disease detection; 10 for healthcare provider support in evaluation, surgery, or telemedicine; six for patient education and raising awareness; and three for promoting regimen adherence among patients. Notably, a relatively high number of apps focused on poor vision or retinal pathologies.

With the ongoing DX in medicine, the clinical utilization of smartphone apps in ophthalmology is anticipated to increase in the near future. However, with the expected spread of mHealth apps, creating universally agreed-upon criteria for evaluating an app's utility, validity, reliability, safety, and usability may be essential to avoid negligent mHealth usage as well as adverse outcomes. With appropriate and careful inspection of mHealth apps, it may be possible to raise the current standard of care in telemedicine and even in routine clinical practice by adding greater intricacy in ophthalmic care.

## Smartphone apps: A new class of SaMD

While various definitions of digital health exist, they intersect in the key aspect of “using ICT in medicine and other health professions to manage illnesses and health risks and to promote wellness”^24)^. Broadly, the realm of digital health encompasses medicine involving wearable devices, AI, mHealth, telemedicine, and personalized medicine^24)^. Digital health is minimally affected by several crucial barriers to conventional healthcare, including geographical and temporal access and cost, thereby promoting the efficacy and cost-effectiveness of medical care^25)^.

**Figure 1:**
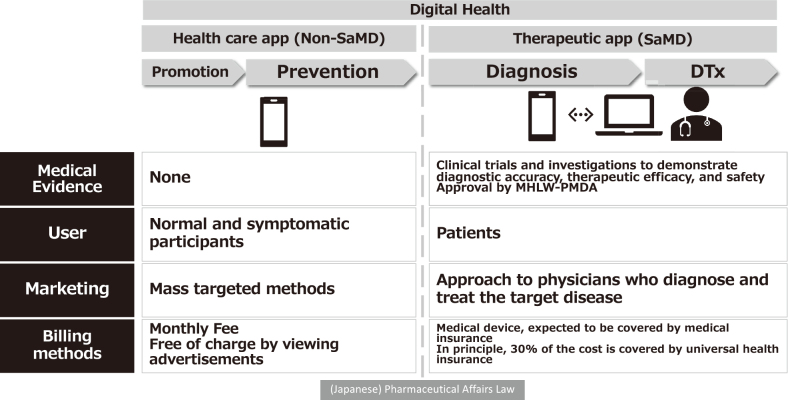
Comparison of healthcare and digital therapeutic apps (SaMD)

Among the countless digital health domains, SaMD refers to software programs developed through robust evidence-driven research, which aim to provide diagnostic, therapeutic, and preventive services. Medical devices based on smartphone apps have gained traction in recent decades. Smartphone apps in medicine can be largely classified into two groups: “healthcare apps,” which promote general health and preventive changes, and “digital therapeutic apps,” which involve treatments or prognostication. However, because of their use as a treatment option for specific diseases, the Pharmaceuticals and Medical Devices Agency of Japan must first review clinical evidence to support their therapeutic use and formally approve their application as medical devices (Figure 1).

Many efforts have been made in Japan to design and develop digital therapeutic smartphone apps to assist in disease treatment, with the CureApp Smoking Cessation app denoting the start of a possible paradigm shift in healthcare as the first-ever app-based service to be recognized as a treatment and reimbursed by the national insurance system in 2020. In November of the following year, SUSMED, Inc.'s (Tokyo, Japan) smartphone app for insomnia treatment assistance also successfully satisfied the inspection criteria of the Ministry of Health, Labor, and Welfare and received regulatory approval.

## Dry eye disease and its unmet medical needs

DED is one of the most prevalent ocular diseases and is thought to affect over 10 billion people globally and 20 million people in Japan alone. Its prevalence is projected to increase in the era of Society 5.0, partially owing to the worldwide trend towards an aging and digital society with its associated lifestyle changes^26-30)^. More importantly, given its chronic nature, its long-term impact on visual quality and work productivity causes economic losses on a global scale^26, 27)^. However, the current first-line approach to DED care is *post facto* symptom management with topical eye drops, and a cure has yet to be developed. Hence, to minimize the impact of DED at a societal level, suppressing its onset and exacerbation before it progresses to severe disease, based on individually tailored preventive and predictive approaches, is crucial for improving the current standard of care^3, 31)^.

DED is highly variable and heterogeneous in terms of presenting symptoms, with complaints ranging from subjective dryness and decreased visual acuity to ocular fatigue and photophobia^32)^, many of which can be considered nonspecific during patient evaluation. Several clinicians have raised a reasonable concern that this may lead to a significant proportion of missed or under-diagnosis, as corroborated by our recent study^12)^ that used a smartphone app developed for DED research, which also suggested that public health intervention may be required. Additionally, even with a formal DED diagnosis, numerous societal barriers to regular healthcare visits, including work and academic responsibilities, in addition to the changes that have followed the COVID-19 pandemic, exist. In populations with activity restrictions, such as older individuals and children, those with physical disabilities, and immunosuppressed individuals, these obstacles to appropriate evaluation and follow- up are even greater.

While the demand for remote DED examination is increasing, the established gold standard for DED diagnosis involves examination under slit-lamp biomicroscopy and techniques that can assess the tear film and corneal status (i.e., tear film break-up time, tear meniscus, and fluorescein cornea staining)^33)^. Remote diagnosis of DED remains an unmet need. In search of an effective solution for this unmet medical need, using the principles of mHealth and smartphone apps, InnoJin Inc., a start-up company with venture backing from Juntendo University, has attempted to develop app-based SaMDs for DED diagnostic support^10)^.

## Research, development, and clinical implementation of DryEyeRhythm^®^: A smartphone app-based dry eye disease diagnostic

**Figure 2:**
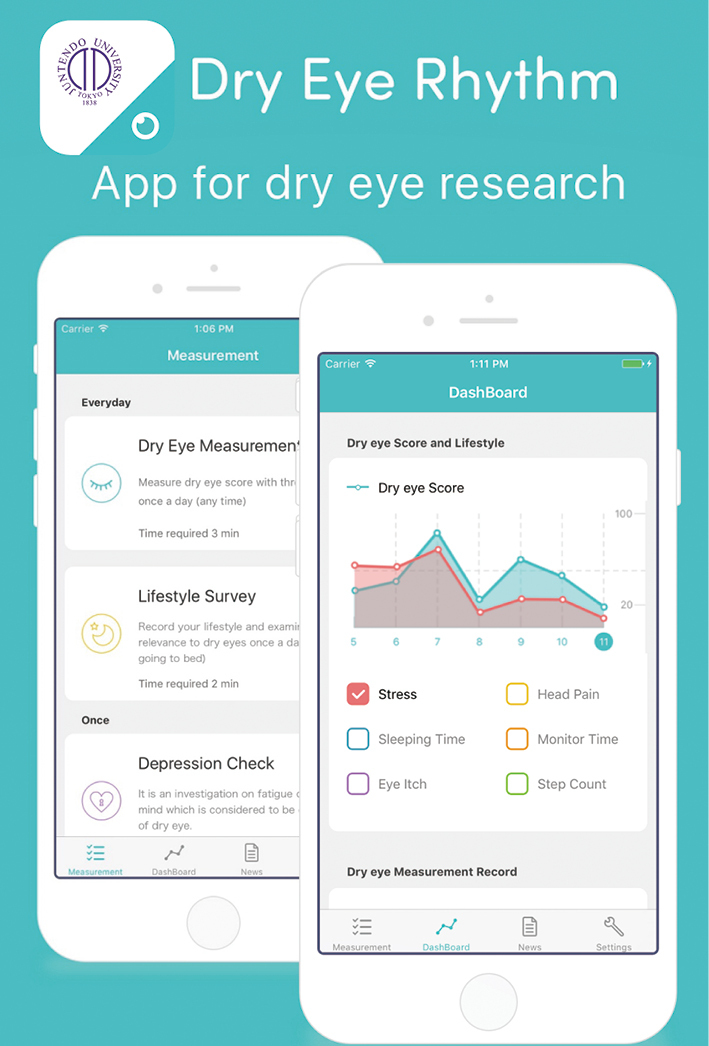
Illustration of DryEyeRhythm^®^, an app for dry eye research

Since 2016, our team has endeavored to design and develop DryEyeRhythm^®^(Figure 2), a smartphone app for DED research. Following its release, DryEyeRhythm^®^ has been implemented in several noteworthy studies, including those related to identifying risk factors for DED exacerbation and those associated with undiagnosed DED, establishing stratification algorithms to improve DED subtype delineation based on symptomologies, and proposing new app-based non-invasive and non-contact diagnostic tools for DED, particularly in light of the recent COVID-19 pandemic^3, 12, 34-37)^. Using the touch screen and front-facing camera of commonplace smartphones, users can easily measure their maximum blink interval^38)^ and complete and submit DED-specific questionnaires (Japanese Version of the Ocular Surface Disease Index)^39)^ for analysis. In a recent validation study, compared with the traditional diagnostic standard of the subjective DED symptom questionnaire plus tear film break-up time, our app-based diagnostics yielded a sensitivity of 71.4%, a specificity of 87.5%, and an area under the curve of 0.910 (0.846-0.973)^36, 40)^. Based on these findings and lessons learnt along the way, we are actively conducting a clinician-initiated investigative clinical study to obtain approval for SaMD using a smartphone application that can assist in DED diagnosis^10)^.

The clinical and social implementation of a smartphone app-based SaMD that can assist in DED diagnosis may have strong implications in several key areas. First, as the penetration of smartphones in our society continues to increase, the dependence on highly specialized tools and facilities (i.e., ophthalmology clinics with slit-lamp biomicroscopes, fluorescein dye, meibography, and keratography^41)^) for formal diagnoses may substantially decrease, and patients may receive more timely care through widely available over-the-counter artificial tears, without delaying treatment until they can be seen in-person. Additionally, patients with barriers to reliable healthcare access will benefit from the capability to receive remote preliminary evaluations and follow-up care through telehealth services prior to an in-person visit.

## Conclusion

As Society 5.0 unfolds, conventional medicine will most likely experience rapid DX, and the rapid implementation of digital health technology will boost the overall value and cost-effectiveness of healthcare. The current unmet medical need for prompt DED diagnosis and uninterrupted monitoring of numerous patients with healthcare barriers can be addressed by DX, to prevent the onset and progression of this disease.

## Funding

This work was supported by Novartis Research Grants 2018 (TI), Daiohs Foundation Research Grants 2018 (TI), JST COI Grant Number JPMJCER02WD02 (TI), JSPS KAKENHI Grant Numbers 20KK0207 (TI), 20K23168 (AMI), 21K17311 (AMI), and 21K20998 (AE), and 22K16983 (AE), the OTC Self-Medication Promotion Foundation (TI and YO); UBE INDUSTRIES FOUNDATION (TI), Charitable Trust Fund for Ophthalmic Research in Commemoration of Santen Pharmaceutical's Founder (TI); TERUMO LIFE SCIENCEFOUNDATION, Kondou Kinen Medical Foundation (TI), Nishikawa medical foundation 2020 (TI); and Takeda Science Foundation 2022 (TI). This study was supported by the following: SEED Co., Ltd.; Alcon Japan, Ltd.; Santen Pharmaceutical Co., Ltd.; Rohto Pharmaceutical Co., Ltd.; HOYA Corporation; WAKAMOTO Co., Ltd. The funders had no role in the design or conduct of the study, collection, management, analysis, or interpretation of data, preparation, review, or approval of the manuscript, or the decision to submit the manuscript for publication.

## Author contributions

TI conceived of the concept of this paper and was major contributor in writing the manuscript. JS was major contributor in writing the manuscript. TI, YO, KN, AMI, AE, KH, YA, HT, and YM contributed to the development of the study protocol and collected the data. TI, YO, AIM and AE performed funding acquisition. All authors reviewed the advanced the concepts within the paper and drafted the manuscript. All authors read and approved the final manuscript.

## Conflicts of interest statement

The DryEyeRhythm application was created using Apple's ResearchKit (Cupertino, CA, USA) along with OHAKO, Inc. (Tokyo, Japan) and Medical Logue, Inc. (Tokyo, Japan). TI reports non-financial support from Lion Corp. and Sony Network Communications Inc.; grants from Johnson & Johnson Vision Care Inc., Yuimedi Inc., ROHTO Pharmaceutical Co. Ltd., Kobayashi Pharmaceutical Co. Ltd., Kandenko Co. Ltd., and Fukoku Co. Ltd.; and personal fees from Santen Pharmaceutical Co. Ltd., InnoJin Inc., and Ono Pharmaceutical Co. Ltd., outside the submitted work. YO, KN, and AMI received personal fees from InnoJin Inc., outside the submitted work. SN report grants from Kowa Co. Ltd.; Mitsubishi Tanabe Pharma Corp.; Alcon Japan, Ltd.; Santen Pharmaceutical Co. Ltd.; Machida Endoscope Co. Ltd.; Wakamoto Pharmaceutical Co. Ltd.; Bayer Yakuhin Ltd.; Senju Pharmaceutical Co. Ltd.; Nippon Boehringer Ingelheim Co. Ltd.; Chugai Pharmaceutical Co. Ltd.; Hoya Corp.; and Novartis Pharma KK, outside the submitted work. The remaining authors have no conflicts of interest to declare.
